# Speech Recognition–Based Dietary Assessment Tool for Older Adults: Validation and Usability Study

**DOI:** 10.2196/81336

**Published:** 2026-05-26

**Authors:** Yoonjee ‍Sung, Soyoung Jung, Hae Jin Kang, So Young Moon, Jee Hyang Jeong, Seong Hye Choi, Eun-Hye Lee, Yoo Kyoung Park

**Affiliations:** 1 Department of Medical Nutrition, Graduate School of East-West Medical Science, Kyung Hee University, Gyeonggi, Republic of Korea; 2 Department of Medical Nutrition, Graduate School of East-West Medical Science AgeTech-Service Convergence, Kyung Hee University, 1732 Deogyeong-daero, Giheung-gu, Yongin-si, Gyeonggi, 17104, Republic of Korea, 82 1062311931, 82 312048118; 3 Department of Neurology, School of Medicine, Ajou University, Gyeonggi, Republic of Korea; 4 Department of Neurology, College of Medicine, Ewha Womans University, Seoul, Republic of Korea; 5 Department of Neurology, College of Medicine, Inha University, Incheon, Republic of Korea

**Keywords:** older adults, speech recording, dietary intake assessment, validity, usability

## Abstract

**Background:**

As populations age globally, accurate and feasible dietary assessment for older adults has become increasingly important. South Korea has already become an “aged society,” with over 14.2% of its population being aged 65 years and older, and is projected to become one of the world’s most rapidly super-aged societies by 2050, with more than 40% of its population in this age group. Similarly, the Asia-Pacific region is experiencing accelerated population aging, with 10 countries classified as “aging societies” (>7% aged ≥65 years), 5 as “aged societies” (>14%), and 11 as “super-aged societies” (>21%) in 2025. Despite the growing need for accurate dietary monitoring in this demographic, nutritional assessment remains challenging due to limitations of conventional methods, compounded by cognitive burden, functional decline, and low literacy. Although various technology-based solutions, including web-based, scanner-based, and mobile tools, have been introduced, challenges related to usability, accuracy, and cost remain unresolved.

**Objective:**

This study aims to evaluate the validity and usability of a speech-based dietary assessment tool as a potential alternative to a traditional pen-and-paper food diary among older adults.

**Methods:**

In a randomized crossover study, adults aged ≥65 years (n=18) completed 2 nonconsecutive 3-day dietary assessment periods using both a speech-recording (SR) method and a food diary (FD). Mean daily nutrient intakes were compared between methods, and agreement was examined using Bland-Altman plots. Usability was assessed after each method using the System Usability Scale (SUS).

**Results:**

Mean daily intakes estimated by SR and FD were similar in magnitude across most nutrients. The paired mean difference in energy intake (FD-SR) was 38.38 (95% CI −176.63 to 253.40) kcal. Across nutrients, mean differences were generally small, and most 95% CIs included zero, indicating limited evidence of a large systematic difference at the group level. The correlation coefficient between SR and FD ranged from 0.178 to 0.907 depending on the nutrient assessed, though CIs were often wide (eg, energy intake; Pearson *r*=0.52, 95% CI 0.05-0.81). Bland-Altman analyses for energy and macronutrients show mean differences close to zero, although substantial individual-level variability was observed. Cholesterol demonstrated greater dispersion and possible proportional bias at higher intake levels. Mean SUS scores were 66.25 for FD and 72.78 for SR, with a paired mean difference (FD-SR) of −6.53 (95% CI −11.75 to −1.30), indicating higher usability ratings for the SR method on average.

**Conclusions:**

These findings suggest that in older adults, the speech recognition method–produced dietary intake estimates may be a feasible approach with broadly comparable nutrient intake levels to written food diaries and modestly higher usability ratings. Further work is needed in larger samples to compare approaches with increased precision.

## Introduction

The demographic landscape of South Korea is undergoing a notable transformation, with projections indicating that by 2060, individuals over 65 years will constitute approximately 43.9% of the total population [1]. This demographic shift underscores the urgent need to address the nutritional needs of older adults. Despite this, malnutrition among older adults has been on the rise since 2014, underscoring the need for feasible routine dietary assessment approaches that accommodate age-related sensory, cognitive, and literacy challenges [2]. Consequently, there is a compelling need for an enhanced dietary assessment tool specifically designed to accurately evaluate the overall nutritional status of the older population.

Nutritional assessment involves evaluating various factors such as anthropometric measurements, biochemical indicators, clinical history, and dietary intake. Accurate documentation and analysis of dietary habits are crucial for diagnosing malnutrition and implementing effective interventions [3]. Conventional methods like self-reported food records and food frequency questionnaires (FFQs) are labor-intensive and impractical for those with varying literacy levels and cognitive impairments [4]. Technological advancements have introduced new dietary assessment methods, such as web-based 24-hour recall systems, mobile food diary (FD) apps, and camera-based sensors [5]. However, these methods face challenges related to accuracy, accessibility, and literacy [6].

Among the new innovative tools available, speech recording has emerged as a promising method for efficient data collection, noted for its simplicity and user-friendliness. This technique enables individuals to capture dietary details through natural speech, thereby reducing participant burden and minimizing the likelihood of omitting essential details [7]. It holds particular value for populations with literacy and sensory impairments, thereby broadening the scope of inclusion to a more diverse demographic [8]. Advances in speech recognition (SR) technology have further automated data processing, enhancing accuracy and reducing the need for manual labor [9].

The SR technology functions by converting audio-recorded speech into text, identifying keywords, and facilitating nutritional analysis [10]. Initially, the SR food search engine converts the audio-recorded speech to text, facilitating the identification of keywords such as the names of consumed foods and their respective quantities. This process aids in the subsequent calculation and analysis of the nutritional value of the meal. With the widespread availability of smartphones equipped with speech-recording capabilities, data collection for speech-based dietary assessment has become more accessible than ever before [11].

With advancements in SR technology, there has been a surge in research interest surrounding the use of speech technology to enhance the process of nutritional assessment, offering a novel approach for a wide range of users [12]. This study, therefore, aims to assess the validity and usability of an SR dietary assessment tool as a viable alternative to the traditional pen-and-paper FD among older adult participants. The study focused on assessing the agreement between the SR and FD methods, validating the accuracy of the SR tool, and examining its practical usability. Due to the impracticality of precisely measuring an individual’s self-reported diet over extended periods, studies often compare new methods with existing dietary assessment tools to assess their relative merits despite their inherent limitations [13].

Unlike many previous validation studies that operationalized dietary intake primarily at the level of individual food items or ingredients, this study evaluates dietary intake using a dish-based reporting framework. Dish-based assessment is particularly relevant in dietary contexts where meals commonly consist of multiple prepared dishes containing mixed ingredients, which can increase the cognitive burden of recalling and reporting individual components. In Korean dietary patterns, meals often include several mixed dishes that are placed centrally and consumed communally within a single eating occasion. Because individuals typically serve variable portions from shared plates, estimating intake at the ingredient level can be especially complex and prone to omission. Dish-level reporting may therefore provide a more pragmatic and cognitively efficient approach to reduce omission and reporting burden in such settings characterized by both mixed ingredient preparations and communal sharing. To our knowledge, no prior validation study has examined SR-based dietary assessment using a dish-based reporting structure, highlighting the need to evaluate its applicability in this context [14].

Therefore, this study aims to evaluate the validity and usability of an SR-based dietary assessment tool in comparison with a traditional pen-and-paper FD among older adults. Specifically, we examined the agreement between the SR and FD methods for estimating dietary intake and assessed the practical usability of the SR tool in this population.

## Methods

### Study Design

**Figure 1.**
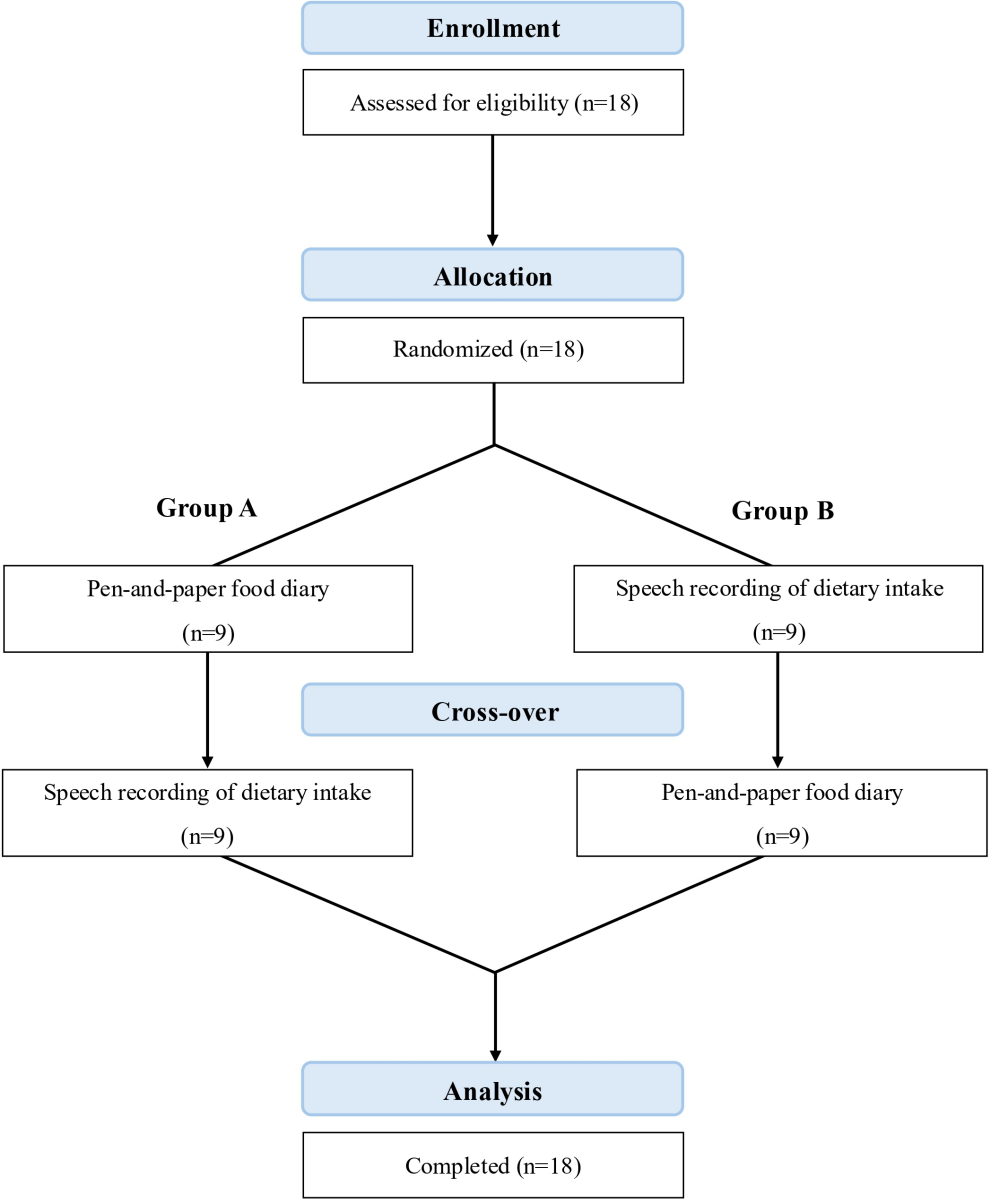
Flowchart of participants.

This study was conducted at Kyung-Hee University from February 2024 to March 2024 after review and approval by the Institutional Review Boards of Kyung-Hee University (No. KHGRIB-24‐040). This study used a randomized crossover (AB/BA) design to compare the effectiveness of 2 dietary assessment methods: the pen-and-paper FD method and the SR method over a 2-week period (Figure 1). Participants were randomly assigned to one of 2 sequences (FD followed by SR, or SR followed by FD) using a computer-generated random sequence created in Microsoft Excel. Each method was used during a nonconsecutive 3-day recording period per week.

### Study Participants

Eligible participants were older adults aged 65 years or older of both genders who owned smartphones and were able to complete 3 nonconsecutive days of dietary recording over a 2-week period. Because the SR tool required independent smartphone use, digital competency was considered an integral component of recording capability. Participants were therefore screened for basic digital literacy proficiencies using a tailored survey comprising 19 questions focused on digital familiarity, digital efficacy, and basic digital skills [15]. Inclusion criteria required scores ≥3 for digital familiarity and efficacy and ≥2 for basic digital skills, indicating sufficient digital competency for independent smartphone-based recording.

Participants diagnosed with mild cognitive impairment or dementia, or with significant visual or hearing impairments, were excluded. Individuals were also excluded if, based on researcher judgment, scheduling factors (eg, extended travel or irregular routines) were likely to interfere with accurate dietary recording.

Participants were recruited via posters, word-of-mouth referrals, and social networking platforms over a one-month period. General characteristics, including age, gender, education level, marital status, living status, comorbidities, and financial status, were collected using structured self-reported questionnaires administered with interviewer guidance. A total of 18 individuals were screened; all met the inclusion criteria, and none were excluded. Sample size was determined using G*Power (Heinrich Heine University Düsseldorf) for a 2-tailed paired *t* test (α=.05; power=0.95), assuming an effect size (Cohen *d*) of 1.04, based on effect sizes reported in previous dietary validation studies [13]. Under these assumptions, a minimum of 14 participants was required. To account for potential dropout, 18 participants were recruited and randomized to assessment order.

An effect size of 1.04 SDs indicates that the study was powered to detect relatively large differences between methods; for example, given the observed variability in energy intake (SD ≈400-500 kcal), this corresponds to a detectable difference of approximately 400‐500 kcal between FD and SR. Smaller differences would therefore be unlikely to be detected with this sample size.

### Data Collection

#### Anthropometric Measurements

Anthropometric data including height, weight, BMI, skeletal muscle mass, body fat percentage, mid–upper arm circumference, and calf circumference were collected. Body composition was measured using the InBody 920 (InBody Co, Ltd), and body measurements such as mid–upper arm circumference and calf circumference were taken with a standard tape measure.

#### Nutritional Status Assessments

The nutritional status of participants was evaluated using the Mini Nutrition Assessment (MNA) and the Nutrition Quotient-Elderly (NQ-E) questionnaires. The MNA assesses malnutrition risks through various criteria, including BMI, weight loss, mobility, psychological stress, and other factors, categorizing scores as normal nutritional status (24‐30 points), risk of malnutrition (17‐23.5 points), and malnourishment (0‐16.5 points) [16]. The NQ-E assesses nutritional status and meal quality through 20 questions on food intake frequency and eating behaviors, with higher scores indicating better nutritional status [17]. Nutritional assessments were conducted to identify discrepancies between nutritional status and nutrient intake, revealing potential validity limitations, such as poor nutritional health despite normal nutrient intake levels or vice versa [18].

#### Dietary Intake Assessments

Dietary intake was assessed using both a pen-and-paper FD and a speech-recording food log. Participants recorded their dietary intake for 3 nonconsecutive days each week using both methods. Nutrient intakes were analyzed using CAN-Pro 6.0 software (Korean Nutritional Society). For both methods, recorded food items and portion sizes were manually entered into CAN-Pro by trained researchers following the same coding procedures.

#### Pen-and-Paper FD

Participants documented their dietary intake manually on provided sheets, detailing the date, time, location, and food items for each meal and snack. Participants were instructed to report portion sizes using standardized household measures or food scales, consistent with the guidance provided for the speech-recording method.

#### SR of Dietary Intake

Participants used their smartphones to verbally record dietary intake, describing foods and portion sizes using standardized household measures or food scales. Audio recordings were transcribed using CLOVA (Naver), the cloud virtual assistant SR engine widely used for transcribing verbal input into text format. Transcription accuracy was validated by comparing machine-generated transcripts with human transcriptions.

For both the FD and SR methods, participants were instructed to photograph their plates before and after consumption. The photographs were collected solely for quality control purposes, such as identifying potentially omitted items (eg, snacks and beverages) and verifying portion sizes. These procedures were applied equally to both methods, and all nutrient analyses and method comparisons were based exclusively on participant-reported data rather than photographic records.

#### Usability Evaluation

The usability of each dietary assessment method was evaluated using the System Usability Scale (SUS) [19]. The SUS includes 10 items on a Likert scale, with scores ranging from 0 to 100, where higher scores indicate better usability. SUS scores were interpreted through acceptability ranges (0‐50 considered “not acceptable,” 50‐70 “marginally acceptable,” and 70‐100 were considered “acceptable”), descriptive adjectives (“Excellent” for scores above 85, “Good” around 71, “OK” around 51, to “Poor” for lower scores), and a grading system from A (superior) to F (failing). Participants completed the SUS questionnaire weekly, after a week of using each dietary assessment method, to provide feedback on user experience.

### Statistical Analysis

All analyses were conducted using SPSS (version 29.0; IBM Corp), for macOS. Normality of data distribution was assessed using the Shapiro-Wilk test. Differences in mean nutrient intakes between the SR and FD methods were summarized using mean differences with 95% CIs. An independent *t* test or Mann-Whitney *U* test was used as a supplementary analysis, where appropriate, to aid interpretation. The sample size for this study was determined based on the primary aim of the intervention trial rather than for the purpose of method comparison. Consequently, the present validity analysis was conducted with a preexisting sample, and the study may have had limited statistical power to detect systematic differences between the SR and FD methods. Therefore, CIs including zero should not be interpreted as definitive evidence of absent bias, but rather as reflecting uncertainty due to limited power.

Pearson correlation coefficients were calculated to assess the linear association between absolute nutrient intake estimates derived from the 2 dietary assessment methods. 95% CIs for Pearson correlation coefficients were calculated using Fisher z transformation. Agreement between SR and FD was further evaluated using Bland-Altman plots to assess bias and limits of agreement across the range of intake and selected macronutrients, as these variables represent primary outcomes in dietary validation studies, exhibit broader intake distributions, and allow clearer evaluation of proportional bias across intake levels. Micronutrient intakes, in contrast, are characterized by substantial within-person variability relative to between-person variability [20]. When intake varies markedly within individuals across measurement occasions, this contributes to greater variability in the paired differences between methods, resulting in wide limits of agreement in Bland-Altman analysis. In this context, broad limits may largely reflect within-person variation rather than disagreement attributable to measurement methods [21]. Accordingly, Bland-Altman plots for micronutrients were considered less informative for evaluating systematic agreement in this context.

Continuous variables are presented as mean (SD), and categorical variables as counts and percentages.

### Ethical Considerations

The study was conducted in accordance with the Declaration of Helsinki and approved by the Institutional Review Board of Kyung Hee University (KHGIRB-24-040 on the 21st of February 2024). All participants were fully informed on the purpose and procedures of the study and provided written consent prior to their participation.

## Results

### Overview

General characteristics of the study population. A total of 18 participants (13 women and 5 men) completed the study, each assessed for 2 nonconsecutive 3-day recording periods (Figure 1).

Participants’ ages ranged from 65 to 85 years, with a mean age of 72 (SD 4.89) years (men: mean 71.80, SD 1.79; women: mean 72.30, SD 5.72). The average BMI was 24.92 (SD 2.70) kg/m². Men had higher skeletal muscle mass (mean 28.8, SD 6.4 kg) than women (mean 20.9, SD 2.7 kg). Regarding education, 6 out of 18 (33%) had university-level education, with a higher proportion of men (60%) than women (23%). All participants were married, with most living with their partners (11/18, 61%). The primary source of living expenses was through salary or pensions of the individual or their partner (all men; 11/13, 84% of women). Most participants, 15 out of 18, felt secure in their ability to afford groceries (83%; Table 1).

**Table 1.** Anthropometrics and general characteristics of study participants (n=18).

Variables		Total	Male (n=5)	Female (n=13)
Age (years), mean (SD)		72.17 (4.89)	71.80 (1.79)	72.30 (5.72)
Height (cm), mean (SD)		159.31 (10.41)	167.0 (11.2)	156.4 (8.8)
Weight (kg), mean (SD)		63.86 (10.01)	72.6 (12.5)	60.5 (6.9)
BMI (kg/m^2^), mean (SD)		24.92 (2.70)	25.9 (2.6)	24.5 (2.7)
Skeletal muscle mass (kg), mean (SD)		23.14 (5.25)	28.8 (6.3)	20.9 (2.7)
Body fat mass (kg), mean (SD)		21.12 (4.85)	20.6 (4.6)	21.3 (5.1)
Body fat percentage (%), mean (SD)		32.21 (6.46)	28.58 (5.55)	34.99 (6.04)
Education level, n (%)				
≤Middle school		5 (27.8)	1 (20.0)	4 (30.8)
High school		7 (38.9)	1 (20.0)	6 (46.2)
≥University		6 (33.3)	3 (60.0)	3 (23.1)
Marital status, n (%)				
Married		18 (100.0)	5 (100.0)	13 (100.0)
Single		0 (0.0)	0 (0.0)	0 (0.0)
Living arrangements, n (%)				
Living alone		1 (5.6)	0 (0.0)	1 (7.7)
Living with a partner		11 (61.1)	4 (80.0)	7 (53.8)
Living with children		6 (33.3)	1 (20.0)	5 (38.5)
Other		0 (0.0)	0 (0.0)	0 (0.0)
Living expenses, n (%)				
Salary or pension		16 (88.9)	5 (100.0)	11 (84.6)
Children or relatives		1 (5.6)	0 (0.0)	1 (7.7)
Governmental support		0 (0.0)	0 (0.0)	0 (0.0)
Part-time job		0 (0.0)	0 (0.0)	0 (0.0)
Other		1 (5.6)	0 (0.0)	1 (7.7)
Food security, n (%)				
Yes, secure		15 (83.3)	5 (100.0)	10 (76.9)
No, not secure		3 (16.7)	0 (0.0)	3 (23.1)

### Nutritional Assessment

The MNA scores indicated normal nutritional status for all participants, with a mean score of 27.06 (SD 2.27; men: mean 26.60, SD 2.27; women: mean 27.23, SD 2.34). The NQ-E score was 62.92 (SD 13.96), higher in male participants (mean 70.79, SD 10.68) than female participants (mean 59.89, SD 14.22). Male participants scored higher across NQ-E subscores in balance (mean 62.02, SD 13.79), moderation (mean 60.28, SD 13.61), and practice (mean 66.94, SD 11.75) compared to female participants (balance: mean 60.98, SD 18.15; moderation: mean 59.59, SD 17.81; practice: mean 55.54, SD 14.51).

### Dietary Intake Assessment

An assessment was conducted to evaluate the accuracy and reliability of the SR tool used to transcribe speech recordings of participants, capturing details such as date, time, and meal type (eg, breakfast, lunch, dinner, and snacks), as well as the name of the dish and estimated portions using either standard household objects or food scales. The mean accuracy of transcription across participants was calculated to be 95.40% (SD 0.02%), indicating a high level of fidelity in transcribing and discerning dietary speech records. Each participant’s speech transcription accuracy was individually assessed for all 3 days of recorded speech, with each day’s transcription compared to manual transcriptions. This analysis process further reinforced confidence in the reliability of the SR tool for dietary intake assessment, and the findings substantiate the viability of using SR technology as a reliable means for capturing and transcribing detailed dietary intake data among older adults.

Table 2 summarizes the mean daily energy and nutrient intakes obtained from the FD and SR methods, together with the paired mean differences (FD-SR), 95% CIs, and the correlation coefficients. For most nutrients, the mean differences were small relative to the between-person variability, and the 95% CIs were wide and generally included zero. The CIs suggest that any systematic difference between methods, if present, is likely to be small at the group level.

**Table 2.** Mean energy, macronutrient, and micronutrient intakes measured by food diary (FD) and speech recording (SR).

Nutrient		FD^a^, mean daily Intake (SD)	SR^b^, mean daily Intake (SD)	Mean difference^c^		Correlation coefficient	
				FD-SR (95% CI)		Pearson *r* (95% CI)^d^	
Macronutrient							
Energy (kcal)		1949.44 (497.59)	1941.69 (385.58)	38.38 (–176.63 to 253.40)		0.518 (0.05 to 0.81)	
Carbohydrate (g)		272.40 (74.42)	254.65 (50.36)	12.05 (–26.41 to 50.52)		0.178 (–0.32 to 0.59)	
Protein (g)		82.84 (16.00)	84.39 (17.05)	1.04 (–4.86 to 6.93)		0.738 (0.44 to 0.89)	
Fat (g)		55.86 (19.98)	56.03 (16.51)	2.56 (–4.98 to 10.10)		0.740 (0.44 to 0.89)	
C: P: F (%)^e^		55.9:17.0:25.8	52.5:17.4:26.0	—^f^		—	
Dietary fiber (g)		34.09 (10.49)	31.53 (7.51)	2.95 (–0.86 to 6.76)		0.671 (0.31 to 0.87)	
Total sugar (g)		42.74 (24.79)	40.57 (19.57)	4.46 (–9.59 to 18.51)		0.163 (–0.33 to 0.58)	
Cholesterol (mg)		261.86 (116.61)	302.18 (69.13)	–18.90 (–82.32 to 44.51)		0.178 (–0.32 to 0.59)	
Minerals (g)		21.35 (3.57)	21.24 (5.05)	0.78 (–1.30 to 2.86)		0.656 (0.29 to 0.86)	
Fat-soluble vitamin							
Vitamin A (μg RAE)		337.99 (90.51)	336.38 (198.82)	–22.48 (–144.83 to 99.87)		–0.146 (–0.57 to 0.36)	
Vitamin D (μg)		0.95 (0.70)	1.31 (0.84)	0.29 (–0.16 to 0.74)		0.057 (–0.42 to 0.50)	
Vitamin E (mg)		19.48 (4.49)	19.97 (2.95)	–0.49 (–2.45 to 1.47)		0.907 (0.76 to 0.96)	
Vitamin K (μg)		241.43 (81.48)	244.34 (81.78)	–1.85 (–82.82 to 86.51)		0.701 (0.37 to 0.88)	
Water-soluble vitamin							
Vitamin C (mg)		86.09 (31.25)	76.26 (16.92)	13.41 (–11.07 to 37.89)		0.416 (−0.07 to 0.73)	
Thiamine (mg)		1.31 (0.17)	1.29 (0.27)	0.00 (–0.11 to 0.10)		0.690 (0.35 to 0.88)	
Riboflavin (mg)		2.12 (1.67)	1.53 (0.23)	0.02 (–0.12 to 0.16)		0.871 (0.68 to 0.95)	
Niacin (mg)		13.03 (1.93)	13.85 (2.90)	–0.82 (–2.04 to 1.06)		0.703 (0.37 to 0.88)	
Vitamin B6 (mg)		0.98 (0.39)	0.63 (0.22)	0.02 (–0.16 to 0.20)		0.653 (0.29 to 0.86)	
Folate (μg DFE)		355.89 (52.68)	352.47 (67.26)	4.96 (–55.52 to 65.45)		0.503 (0.02 to 0.80)	
Vitamin B12 (μg)		3.29 (1.85)	2.99 (3.26)	2.58 (–3.19 to 8.35)		0.498 (0.01 to 0.80)	
Minerals							
Calcium (mg)		653.20 (202.81)	644.32 (210.43)	12.08 (–87.60 to 111.75)		0.469 (–0.04 to 0.77)	
Phosphorous (mg)		1408.69 (551.93)	1254.29 (295.47)	24.49 (–69.18 to 118.16)		0.763 (0.49 to 0.90)	
Sodium (mg)		4145.07 (1403.47)	4305.08 (1327.20)	229.02 (–308.89 to 766.93)		0.577 (0.14 to 0.83)	
Potassium (mg)		3350.30 (1136.41)	3263.26 (791.54)	157.33 (–275.52 to 587.19)		0.449 (–0.05 to 0.76)	
Magnesium (mg)		342.23 (110.26)	326.51 (96.59)	28.10 (–6.35 to 62.54)		0.709 (0.38 to 0.88)	
Iron (mg)		14.76 (2.73)	15.44 (4.02)	–0.83 (–2.42 to 0.75)		0.568 (0.13 to 0.83)	

a FD: food diary.

b SR: speech recording.

c Mean difference represents the paired absolute difference between FD and SR (FD-SR), calculated from participant-level mean intakes (n=18), 95% CIs derived from paired *t* tests.

d 95% CIs for Pearson r were calculated using Fisher z transformation.

e C:P:F represents the mean percentage contribution of carbohydrate, protein, and fat to total energy intake, respectively; these values are presented as descriptive energy distribution ratios rather than mean (SD) values.

f Not applicable.

For energy intake, the FD and SR methods yielded mean values of 1949.44 (SD 497.59) kcal and 1941.69 (SD 385.58) kcal, respectively, with a paired mean difference of 38.38 kcal (95% CI −176.63 to 253.40). For carbohydrate intake, the mean difference was 12.05 g (95% CI −26.41 to 50.52 g), while protein and fat showed mean differences of 1.04 g (95% CI −4.86 to 6.93) and 2.56 g (95% CI −4.98 to 10.10). Across micronutrients, mean differences were generally modest in magnitude, although the corresponding CIs were wide, reflecting variability across participants.

Overall, the observed differences are compatible with small overestimation or underestimation by SR relative to FD, rather than indicating a clear systematic shift in one direction. The direction of the paired difference varied across nutrients, with some nutrients showing higher mean intakes with FD and others with SR.

The Pearson correlation coefficient between the 2 methods ranged from 0.178 to 0.907, with corresponding 95% CIs indicating substantial uncertainty for weaker associations (Table 2). Correlations were moderate to high for several macronutrients, including fat (*r*=0.740), protein (*r*=0.738), and dietary fiber (*r*=0.671), as well as for selected micronutrients such as vitamin E (*r*=0.907) and riboflavin (*r*=0.871). For other nutrients, including carbohydrate, total sugar, cholesterol, and certain vitamins, correlations were weaker.

**Figure 2.**
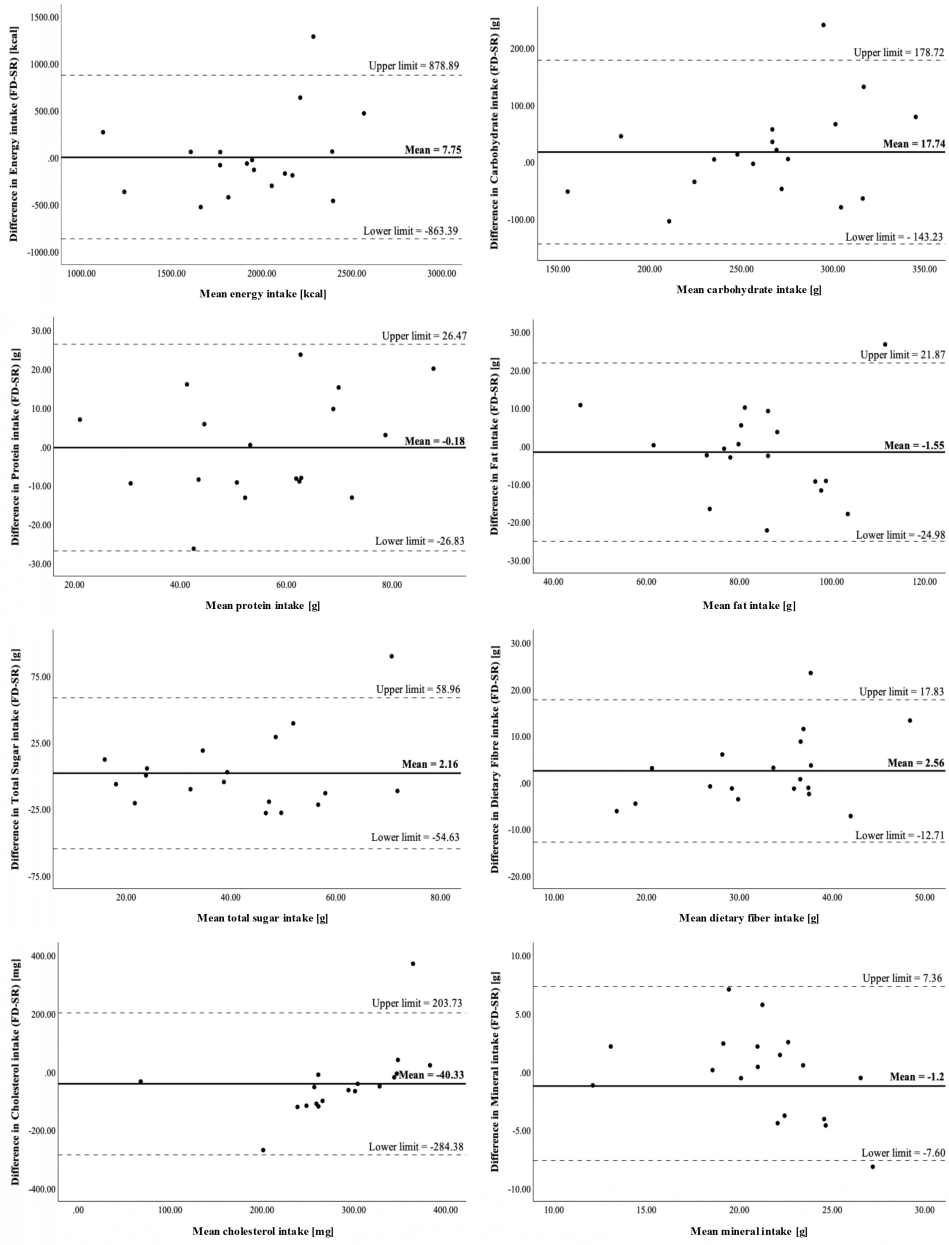
Bland-Altman plots comparing food diary (FD) and speech recording (SR) for energy and selected nutrients. Bland–Altman plots of the difference between intakes recorded by the FD and the SR method against the mean intakes for the 2 reporting methods for energy, carbohydrate, protein, fat, total sugar, dietary fiber, cholesterol, and mineral.

Bland-Altman plots for energy and macronutrients (Figure 2) were used to further examine agreement between FD and SR. For energy intake, the mean difference was 38.38 kcal, with 95% limits of agreement ranging from −863.39 to 878.89 kcal. For fat and protein, mean differences were small relative to the limits of agreement, which were wide. These findings indicate substantial individual-level variability in the differences between methods, consistent with the width of the CIs reported in Table 2. For cholesterol, the plots suggested a possible proportional bias, with larger discrepancies between FD and SR at higher average intakes; however, variability remained considerable across the intake range.

### Usability Evaluation

The SUS scores were obtained from all 18 participants after they had used both dietary assessment methods. The mean SUS score was 66.25 (SD 11.83) for the pen-and-paper FD method and mean 72.77 (SD 10.77) for the SR tool (0‐100 scale). The paired mean difference (FD-SR) was −6.53 (95% CI −11.75 to −1.30), indicating higher usability ratings for the SR method on average.

**Figure 3.**
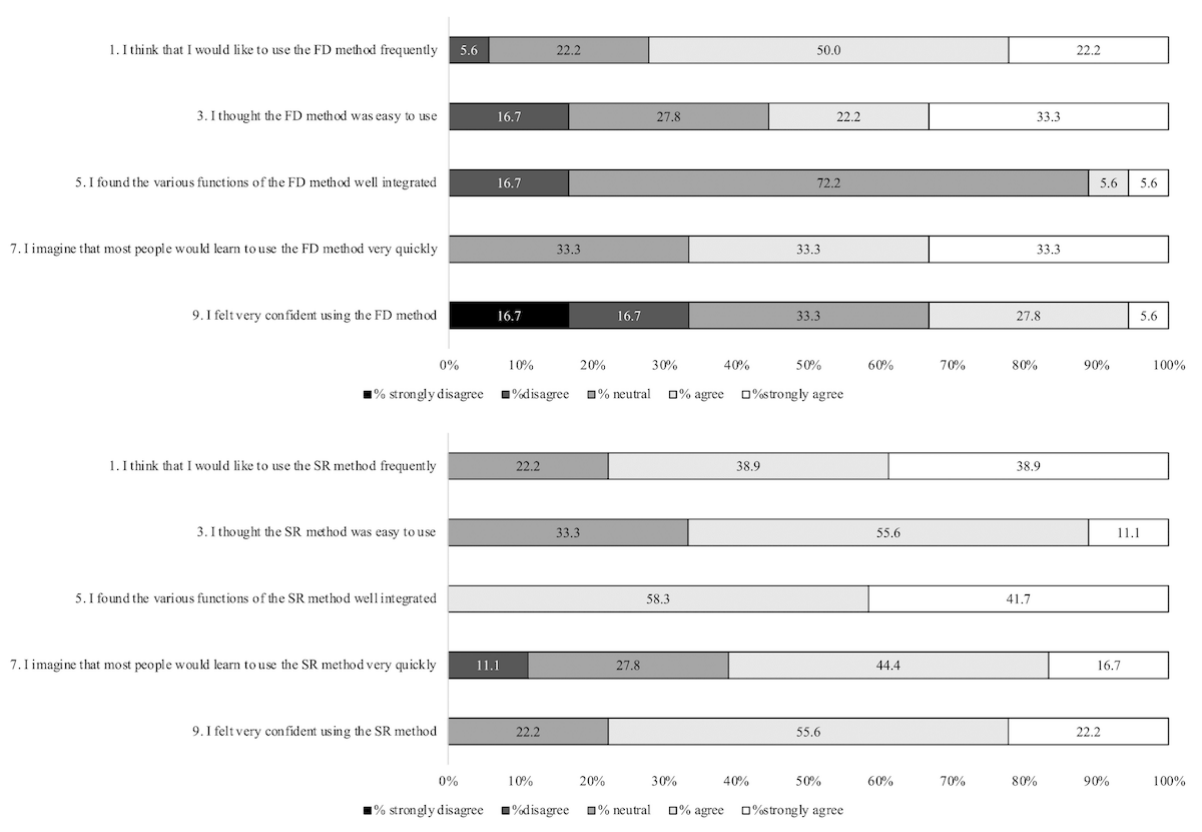
Distribution of the response to System Usability Scale (SUS) items (odd) after completion of food diary (FD) and speech recording (SR). FD: food diary; SR: speech recording.

Responses to individual SUS items are summarized in Figures3  4. Item-level results are presented descriptively as the proportion of participants selecting each response option. Overall, a higher proportion of participants agreed with positively worded statements for the SR method, whereas responses for the FD method more frequently reflected perceived difficulty or the need for support. No inferential statistical testing was performed for individual SUS items, as these results were intended to illustrate response patterns rather than formally compare item-level differences.

**Figure 4.**
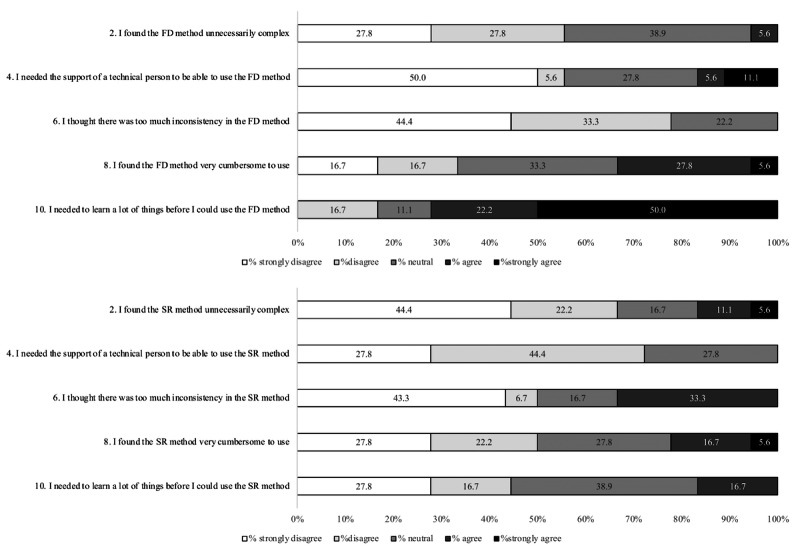
Distribution of the response to System Usability Scale (SUS) items (even) after completion of food diary (FD) and speech recording (SR). FD: food diary; SR: speech recording.

## Discussion

### Principal Findings

This study evaluated the relative validity and usability of an SR dietary assessment method compared with a traditional pen-and-paper FD among older adults. Overall, SR produced dietary intake estimates that were broadly comparable to FD at a group level. Paired mean differences for energy and most nutrients were small in absolute magnitude, and the corresponding 95% CIs generally included zero; however, this should not be interpreted as evidence of absent bias, as the wide intervals reflect substantial uncertainty and limited statistical power to detect systematic differences between methods. Correlation coefficients ranged from 0.178 to 0.907 depending on the nutrient assessed, with moderate associations observed for energy (*r*=0.518), protein (*r*=0.738), fat (*r*=0.740), and dietary fiber (*r*=0.671). Bland-Altman analyses show mean differences close to zero for energy and macronutrients, although limits of agreement were wide, indicating substantial individual-level variability. In terms of usability, SR received higher ratings than FD, with a paired mean difference in SUS scores of −6.53 (95% CI −11.75 to −1.30), indicating a modest but consistent improvement in user experience. Together, these findings suggest that SR may serve as a feasible alternative modality for dietary data entry in older adults, while acknowledging variability at the individual level.

While most previous validation studies have focused on web-based 24-hour recalls, online food frequency questionnaires, and artificial intelligence (AI)–based image recognition tools, this research evaluates SR as an alternative modality for dietary data input, with the potential to simplify data recording and reduce respondent burden [5].

To facilitate comparison with prior validation studies that report relative differences, absolute mean differences were also expressed as percentages of the FD mean intake. When calculated in this way, the relative differences for most macronutrients were below 5%, with the relative difference in energy intake below 1%. These values are comparable to those reported in previous systematic reviews of web-based versus conventional dietary assessment methods, which observed mean energy intake differences of approximately 5.3% (range 0.6%‐16.1%) [5]. Similarly, relative differences for protein and fat (1.84% and 0.32%, respectively) were smaller than those reported in the reviewed studies (5.00% and 7.73%, respectively) [22].

According to the classification proposed by Luevano-Contreras et al [23], relative mean differences of 0.0%‐10.9% are considered “good,” 11.0%‐20.0% as “acceptable,” and >20.0% as “poor.” Interpreted within this framework, most nutrients in the present study fell within the “good” agreement range. The direction of the paired differences varied across nutrients, and the magnitude of these differences was small relative to between-person variability, as reflected in the SDs and CIs.

The Bland-Altman analysis indicated mean differences close to zero for most nutrients; however, limits of agreement were wide, reflecting substantial individual-level variability. Carbohydrate intake showed larger dispersion despite modest mean differences, while fat and protein intake differences were small and consistent across intake levels. In contrast, cholesterol demonstrated proportional bias, with increasing divergence between the 2 methods at higher intake levels, reflected in a funnel-shaped distribution. The limits of agreement for cholesterol were also wider than those reported in previous studies, indicating greater variability between the 2 methods at higher consumption levels.

Correlations between FD and SR indicated moderate linear association for total energy intake (*r*=0.518), essential nutrients (*r*=0.478), vitamins (*r*=0.559), and minerals (*r*=0.589). To aid interpretation, correlation magnitudes were compared with commonly used descriptive thresholds [24]; however, given the small sample size, these values should be interpreted cautiously and viewed as indicative of relative association rather than definitive strength. CIs for several correlation coefficients were wide and, in some cases, crossed zero, reflecting limited precision and statistical uncertainty due to the modest sample size. Certain dietary parameters, including carbohydrates, total sugar, cholesterol, and vitamins A, D, C, and potassium, exhibited weaker correlations, likely reflecting the wide food sources of these nutrients and high day-to-day intake variability [25-28].

Previous studies have similarly reported low correlations for vitamins and minerals in dietary assessments. Kim et al [29] reported correlation of 0.18 for vitamin A and 0.48 for vitamin C using comparable dietary assessment approaches, which aligns with the present findings of −0.146 and 0.416, respectively. These findings highlight the inherent difficulty of capturing micronutrient intake over short recording periods due to underreporting and variability in food consumption [29]. Indeed, Kwon et al [30] estimated that 25‐29 days of dietary records may be required to estimate vitamin intake within 20% of true usual intake with 90% confidence, underscoring a known limitation of short-term dietary recording rather than a method-specific shortcoming.

Usability was assessed using the SUS following completion of both assessment methods. On average, the SR method scored 72.77 (SD 10.77), while the FD method scored 66.25 (SD 11.83). The paired mean difference (FD-SR) was −6.53 (95% CI −11.75 to −1.30), indicating higher usability ratings for SR on average. Although the magnitude of the difference was modest, it is consistent with a perceptible improvement in user experience. According to Bangor et al [31], SUS scores of 68 or above are generally interpreted as “good” and “acceptable” regarding usability, placing SR within this range, while FD fell slightly below this threshold. Previous research evaluating web-based dietary recall systems, such as the Automated Self-Administered 24-Hour Dietary Assessment Tool (ASA24) [32] and EatWellQ8 [33], a food frequency questionnaire system, and other mobile dietary intake applications [34], reported SUS scores ranging from 58 to 75 points, aligning with the SR method’s score.

Patterns of individual SUS item responses suggest that participants perceived SR as easier to learn and more convenient for routine use, whereas FD was more frequently associated with perceived complexity and the need for support. These findings likely reflect the burden of immediate documentation and the cognitive demands of estimating ingredients and quantities in traditional food records [35]. Nonetheless, some participants reported initial difficulty with the SR system, potentially due to unfamiliarity with digital tools, suggesting that training and system refinement remain important for wider implementation.

The randomized crossover design minimized sequence bias and strengthened internal validity by allowing each participant to serve as their own control [36]. Furthermore, the high transcription accuracy of the SR tool underscored its precision in transcribing dietary data, thereby supporting the technical feasibility of the SR system. However, dietary intakes were assessed over only 3 days per method, which limits the precision of micronutrient estimation and may amplify day-to-day intake variability [37,38].

In addition, the small sample size limits statistical power and means that only relatively large differences between methods could be detected. This also precluded subgroup analyses by demographic characteristics (eg, age, sex, education level, or digital literacy), which may influence dietary reporting behavior and usability perceptions. Future studies with larger and more diverse samples are needed to examine these factors in greater detail.

Importantly, this study evaluated relative validity against the traditional pen-and-paper FD method, which itself is subject to recall bias and reporting error. In the absence of an objective gold standard for habitual intake, the findings should be interpreted as evidence of comparability, not absolute accuracy [13]. This SR workflow also required it. Manual postprocessing following transcription, highlighting the need for future fully integrated conversational AI systems capable of real-time prompting and nutrient analysis.

### Conclusion

This study evaluated the relative validity and usability of a novel speech-recognition-based dietary assessment tool compared with a traditional pen-and-paper FD among older adult participants. The findings indicate that SR produces dietary intake estimates that are broadly comparable to FD across most nutrients, with small mean differences, moderate correlations, and acceptable agreement in Bland-Altman analyses. Usability ratings were higher for the SR method, with a modest paired mean difference favoring SR.

Rather than demonstrating superiority, these results support the feasibility of SR as an alternative dietary data entry modality for older adults. With further refinement toward fully integrated, conversational dietary assessment systems, speech-based approaches may offer practical advantages for reducing user burden while maintaining acceptable data quality. Future studies should evaluate performance over longer recording periods and examine the capacity of SR-based systems to accurately identify nutritionally vulnerable individuals in real-world settings.

From an implementation perspective, the SR-based dietary assessment evaluated in this study can be applied in aging and aged societies using the available smartphone technology at the time of this writing. Because the method relies on natural speech input rather than written recording, it may reduce documentation burden and support dietary data collection among older adults with visual, literacy, or dexterity limitations. In practice, such an approach could be used for community-based nutrition monitoring, longitudinal dietary surveys, or remote dietary data collection in public health and research settings. With further development toward automated nutrient analysis and integrated conversational prompting, speech-based systems may complement existing dietary assessment tools while maintaining acceptable data quality. However, broader application will require validation in larger and more diverse older adult populations.

## Abbreviations

AI: artificial intelligence
ASA24: Automated Self-Administered 24-Hour Dietary Assessment Tool
FD: food diary
FFQ: food frequency questionnaire
MNA: Mini Nutritional Assessment
NQ-E: Nutrition Quotient for the Elderly
SR: speech recognition
SUS: System Usability Scale
