# Correction: Speech Recognition–Based Dietary Assessment Tool for Older Adults: Validation and Usability Study

**DOI:** 10.2196/104170

**Published:** 2026-07-16

**Authors:** Yoonjee ‍Sung, Soyoung Jung, Hae Jin Kang, So Young Moon, Jee Hyang Jeong, Seong Hye Choi, Eun-Hye Lee, Yoo Kyoung Park

**Affiliations:** 1 Department of Medical Nutrition, Graduate School of East-West Medical Science, Kyung Hee University, Gyeonggi, Republic of Korea; 2 Department of Medical Nutrition, Graduate School of East-West Medical Science AgeTech-Service Convergence, Kyung Hee University, 1732 Deogyeong-daero, Giheung-gu, Yongin-si,Gyeonggi, 17104, Republic of Korea, 82 1062311931, 82 312048118; 3 Department of Neurology, School of Medicine, Ajou University, Gyeonggi, Republic of Korea; 4 Department of Neurology, College of Medicine, Ewha Womans University, Seoul, Republic of Korea; 5 Department of Neurology, College of Medicine, Inha University, Incheon, Republic of Korea

In “Speech Recognition–Based Dietary Assessment Tool for Older Adults: Validation and Usability Study” [1], the authors noted one error.

The corresponding author’s name appeared as:

Yoo Kyung Park

The name has been changed to:

Yoo Kyoung Park

The correction will appear in the online version of the paper on the JMIR Publications website, together with the publication of this correction notice. Because this was made after submission to PubMed, PubMed Central, and other full-text repositories, the corrected article has also been resubmitted to those repositories.
